# Evaluating the effects of simulated interprofessional teaching on the development of clinical core competence in nursing students: a mixed methods study

**DOI:** 10.1186/s12912-022-01108-5

**Published:** 2022-12-19

**Authors:** Xin-yi Zhou, Yan-feng Wang, Chun-xia Dou, Xiao-ying Tian, Jin Su, Yan-ya Chen, Feng-xia Yan, Qiao-hong Yang, Wenru Wang

**Affiliations:** 1 School of Nursing, Jinan University, Guangzhou, China; 2 Alice Lee Centre for Nursing Studies, Yong Loo Lin School of Medicine, National University of Singapore, Singapore, Singapore

**Keywords:** Simulated interprofessional education, Interprofessional cooperation, Critical thinking, Medical students

## Abstract

**Background:**

While single-method studies have reported on the effectiveness of simulated interprofessional teaching, our understanding of its full effects remains incomplete. Teaching design also provides no relevant theoretical guidance, which reduces the scientific quality and rigor of research. The purpose of this work was to study the effects of the simulated interprofessional education (SIPE) teaching model based on the 3P theory on the course of "Clinical Critical Thinking Training" through a convergent mixed method, and to provide the basis for future teaching design.

**Methods:**

A convergent mixed-method design was used, which consisted of a survey and a semi-structured interview. Data collection took place from September 2021 to July 2022. A cluster sampling method was used to select 60 full-time nursing students from a school in China, and randomly divide them into a control group of 36 and an experimental group of 24. According to the principle of voluntary participation, 6 students majoring in clinical medicine and 6 students majoring in pharmacy were recruited to join the experimental group to form an interprofessional team. The students studied “Clinical Critical Thinking Training” together, in which the control group used traditional simulation teaching and the experimental group used SIPE. The CCTDI (California Critical Thinking Disposition Inventory) and AITCS-II Student (Assessment of Interprofessional Team Collaboration in Student Learning Scale) were used for quantitative evaluation before and after the course, and descriptive statistics and Mann–Whitney U test were used to compare the critical thinking and interprofessional collaboration skills of the two groups of students. Semi-structured interviews were used for qualitative evaluation. Thematic analysis was used to understand student development on the basis of inter-professional core competencies and learning experience.

**Results:**

The students’ interprofessional cooperation abilities and critical thinking scores improved compared with the beginning of the course, but the scores of the experimental group were significantly higher than the control group (*p* < 0.05). Three themes emerged regarding simulated interprofessional teaching: clarifying team positioning, improving team efficiency, and optimizing the learning experience.

**Conclusion:**

SIPE can build students' critical thinking, teamwork, and interprofessional core competencies, which makes it a useful teaching design.

## Background

Simulation refers to replacing or amplifying real experience by evoking or replicating important aspects of the real world in a fully interactive way [1]. Simulation teaching has been found to provide learners with an immersive and realistic teaching mode [2], meet the needs of students for repetitive learning, and find mistakes and learn lessons through personalized learning [3]. Compared with traditional teaching methods, the simulation teaching method can effectively improve clinical core competencies such as critical thinking ability, but is less effective at building cooperative ability [4]. The ability to cooperate is crucial to the success of a clinical practice, but current simulation training is mainly based on a single role-playing model, which does not permit cooperation between various disciplines and cannot establish an interprofessional role identity [5].

Patient recovery and the health care system as a whole cannot function without cooperation between the various health care disciplines. Collaborative care is the gold standard model for healthcare that aims to best assist patients through interdisciplinary collaboration [6]. Interprofessional education refers to how two or more professionals understand, learn, and collaborate to improve patient health outcomes [7]. Studies have shown that interprofessional education can enhance teamwork learning, improve the quality of patient care [8], shorten patient hospital stays, and improve medication safety [9]. Due to the needs of medical personnel training, recent works [10, 11] explored the effects of the combination of simulation teaching and interprofessional education in the form of a simulated interprofessional education model to cultivate critical thinking and teamwork ability. Meyer et al. [12] found that nursing students can improve their pharmacology learning, increase their understanding of other professional roles, and develop interprofessional communication skills by simulating an interprofessional education model. Banks et al. [13] found that teaching students using a simulated interprofessional education model can improve their confidence in simulated collaborative situations and apply the knowledge, skills, and learned values to improve patient safety and health outcomes [14]. Although a simulated interprofessional education model has been implemented in the United States, Europe, and other Western countries [15–17] with good teaching results, it lacks the guidance of systematic theory requires more rigorous teaching methods. In addition, single-method study has limited interpretation of the results and is not conducive to improving teaching methods.

Biggs [18] proposed the learning process 3P theory—prediction, process, and product—as a way to promote critical awareness, emphasize more intelligent and timely decision-making, and improve comprehensive ability [19]. Although there have been study [20] that have applied this theory to interprofessional simutation teaching and promoted the complementarity and integration between medical professions, a problem with this teaching process is that results obtained from a single quantitative study cannot clarify the reasons for such outcomes and improve instructional design Cunningham et al. [21] showed that a mixed research method that combines qualitative and quantitative methods can result in complementary advantages, better understanding of the educational process from different perspectives, improve student participation, promote a better understanding of research questions, and improved teaching strategies. However, in China and other Asian countries, studies using mixed research methods to design and evaluate models of interprofessional collaborative simulation teaching are insufficient. Therefore, in order to ensure a reasonable level of education and meet the high-quality training needs of medical and health personnel, it is important to explore the effects of innovative education models [22].

This study aimed to use the 3P theory to guide interprofessional simulation teaching and adopt a mixed research method to test the effects of a simulated interprofessional education model on the critical thinking and cooperative abilities of students in a "Clinical Critical Thinking" course. This paper further hopes to provide a reference for future teaching models in China and other Asian countries.

### Problem statement

- 1. How is the SIPE model designed?
- 2. How effective is the SIPE model at improving critical thinking level, teamwork ability, and inter-professional core competence?
- 3. What are the students’ learning experiences within the SIPE model?

## Methods

### Design

**Fig. 1:**
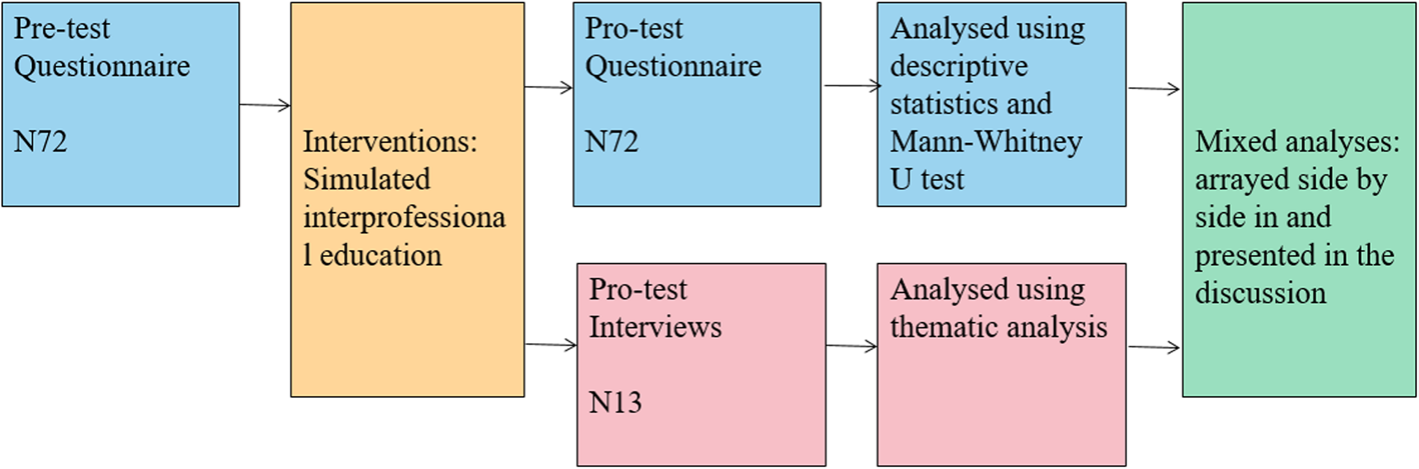
Study overview

The convergent mixed-method design included a questionnaire for quantitative data and semi-structured interviews for qualitative data. The rationale for choosing a mixed-methods design was the recognition of a need for different methods that, in combination, can provide a better understanding of the impact of SIPE on instructional outcomes [21, 22]. This study was approved by our university's ethics committee. Figure 1 provides an overview of this study.

### Participants

A cluster sampling method was used to select 60 full-time third-year nursing students in a school in China and randomly divide them into a control group of 36 students and an experimental group of 24 students. Based on the principle of voluntary participation, 6 clinical medicine major students and 6 pharmacy major students were also recruited into the experimental group. Inclusion criteria: (1) Completion of the corresponding basic medical and professional courses by following the professional training plan and passing the course assessment. (2) Informed consent. Exclusion criteria: Those with incomplete data collection.

### Setting and intervention

Applying SIPE to the “Clinical Critical Thinking Training" curriculum aimed to develop students' clinical critical thinking and improve their abilities to work as a part of interprofessional teams. The specific goal of the intervention was for students to use teamwork to identify patients' problems, perform effective assessments, make the correct diagnosis, and take effective corrective measures to improve patient care.

**Fig. 2:**
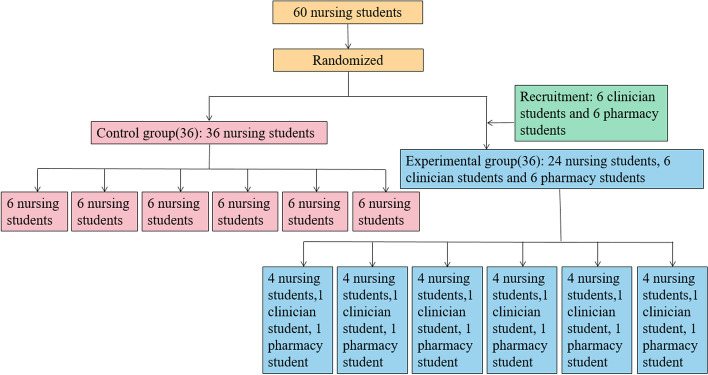
Research group

“Clinical Critical Thinking Training” is the core nursing course at our school. It is 2 credits and includes 28 h of theoretical courses and 20 h of experimental courses, for a total of 48 h. The experimental class is 4 h per session, held once a week for 5 consecutive weeks. The simulation case is designed by nursing, clinical, and pharmacy teachers. Case scenarios focused on patients in the medical, surgical, and intensive care units. The same teaching materials, simulation cases, and instructors were used in the two groups. To permit every student to participate in the course and play an irreplaceable role in the course, each case has six necessary roles: one doctor, one responsible nurse, one auxiliary nurse, one clinical pharmacist, one family member of the patient, and one observer. A total of 12 teams were formed within the control and experimental groups with six members each. The responsible nurse was the team leader, and was responsible for the organization, coordination, division of labor, and cooperation within the team. The control group used conventional simulation teaching, in which nursing students acted as the physicians and clinical pharmacists. The experimental group adopted SIPE, so the roles of physicians and clinical pharmacists were performed by clinical and pharmacy students, respectively. Figure 2 depicts research groupings.

**Fig. 3:**
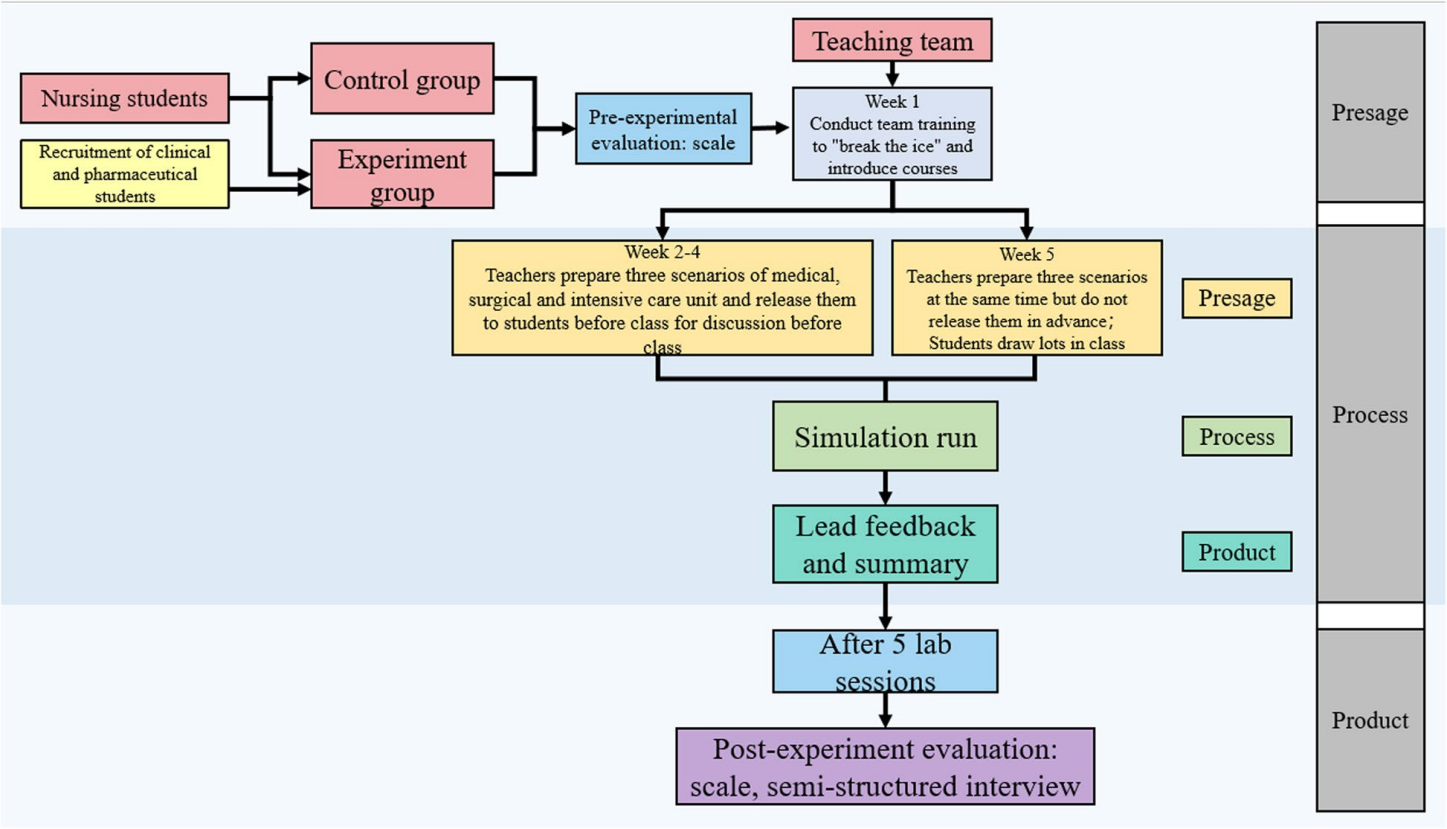
SIPE program design framework

This used the Biggs 3P model as its theoretical framework and was divided into three parts: learning preparation (presage), learning process (process), and learning results (product). The SIPE program design framework is shown in Fig. 3.

Before each class, the inter-professional teaching team needed to create simulated cases and set up situational problems and students needed to review relevant theoretical knowledge and operational skills. Each case included three scenarios: health assessment upon admission, assessment of the implementation of the diagnosis and treatment plan, and health guidance upon discharge. Each scenario included 5–10 min of preparation before the simulation and 15–20 min of case simulation. According to the requirements of the scene simulation, the time ratio of simulation to guidance feedback was 1:2 or 1:3 [23], with a feedback time of 30–40 min. Over the course of the simulation, team members performed a comprehensive assessment of the patient from the perspective of their own specialty based on the case scenario and situational issues, shared the results of that assessment, and jointly formulated examination, treatment, and nursing plans. The whole simulation process was recorded and the instructor did not provide any hints but can adjust the situation at random according to the simulation.

After each simulation, the teacher guided students in their reflection. First, students were asked about the experience of the simulation teaching so that they could fully express and share their emotions. Second, the students watched a video that focused on the key scenarios, and were asked open questions such as “What do you think of what happened,” “How do you feel about it,” and “What will you do next time”. Third, students summarized their learning experience by reviewing key learning points and answering questions about relevant knowledge. The instructor then evaluated each student’s personal performance and the team’s overall performance, focusing on clinical critical thinking and team communication and cooperation as well as nursing specialty students’ cognitive skills, emotional attitudes, communication strategies, and cooperative attitudes. Finally, the instructors pointed out the advantages and drawbacks of the student’s performance and offered suggestions for improvement.

### Data collection

#### Questionnaire

The California Critical Thinking Disposition Inventory (CCTDI) was developed by Facione et al. [24]. Seven aspects of critical thinking are measured by the CCTDI: truth-seeking, open-mindedness, systematicity, self-confidence, inquisitiveness, maturity, and analyticity. The Chinese version of the CCTDI has a total of 70 items [25] and uses a 6-point Likert scale that ranges from 1 (strongly agree) to 6 (strongly disagree). Total scores range from 70 to 420. A score of 40 or more indicates an upbeat personality on that scale and a total score of 280 or above represents positive general critical thinking tendencies.

The Assessment of Interprofessional Team Collaboration in Student Learning Scale (AITCS-II Student) was developed by Orchard et al. [26] based on the first version of the Assessment of Interprofessional Team Collaboration Scale (AITCS). It uses a 5-point Likert scoring method: "never" = 1, "rarely" = 2, "occasionally" = 3, "most of the time" = 4, and "always" = 5. The total score ranges from 17 to 85 points, with an average score ≥ 4 indicating good inter-professional cooperative learning ability [27]. All students in the control and experimental groups completed the CCTDI and AITCS-II before and after the simulation.

#### Semi-structured interview

The Interprofessional Education Collaborative [28] defines interprofessional core competencies in five themes: roles and responsibilities, ethical practice, conflict resolution, collaboration and teamwork, and communication, and recommends that one or more of these themes should be considered when designing interprofessional activities. To understand the effects of the intervention on interprofessional core competencies, after reviewing the relevant literature and clarifying our research questions we designed an interview outline. Following pre-testing and modification by a nursing professor and a medical anthropology professor, the interview outline was finalized and included the following questions: (1) How do you view each role within the team and its responsibilities? (2) Have you meet the standard of professional practice which is expected of your role and fulfilling its responsibilities? (3) Have you encountered disagreements in achieving your team's work goals? If so, how was it resolved? and (4) Talk about your response to the course, such as your gains, lack of interprofessional case design, and suggestions for course improvement.

Interviewees were selected from the experimental and control groups using the purposeful sampling method to ensure a diversity of viewpoints. All interviews were conducted in a separate and quiet conference room at the school. The interviewer had no relationship with the students and was not involved in the development or implementation of the teaching plan. The interviewer explained the purpose of the interview to the students and promise to protect their privacy. The interviewer recorded the entire interview process with the consent of the students. The researcher did not interrupt or comment on the students' statements and observed and recorded the students' expressions and body movements. Before the end of the interview the students were asked if they had any additions and clarifications. Interview recordings were recorded verbatim. The sample size was based on the principle that no new information points appearing during the interview represented data saturation.

### Data analysis

SPSS 26.0 software (IBM, Armonk, NY, USA) was used for descriptive statistics and the Mann–Whitney U test for statistical analysis, with significance defined as α = 0.05. Semi-structured interviews used thematic analysis to process data. Two researchers read the interview content repeatedly and carefully, extracted important sentences, compared similarities and differences, looked for similar meanings or concepts, and formed themes. The integrated themes were fed back to the research subjects for verification, and the themes were defined through group discussions. In a mixed analysis, the results of the two separate analyses were discussed together and were rehearsed side by side, which was consistent with the approach proposed by Creswell [29, 30].

## Results

### CCTDI and AITCS-II Results

The mean critical thinking and interprofessional cooperation scores before and after the experiment are shown in Table 1. No significant differences were observed in critical thinking and inter-professional cooperation between the control and experimental groups before the simulation. Both scores were increased in both the control group and the experimental group after the simulation, but the experimental group scores were significantly higher than the control group (*p* < 0.05).

**Table 1:** AITCS-II and CCTDI at baseline and post-intervention

	CCTDI		Z	*P*	AITCS-II Student		Z	*P*
	Control	Experimental			Control	Experimental		
Pre	237.06 ± 33.44	237.00 ± 28.34	-0.44	0.663	3.60 ± 0.39	3.76 ± 0.36	-1.86	0.063
Post	245.11 ± 30.56	284.08 ± 15.55	-5.81	< 0.01	3.64 ± 0.42	4.03 ± 0.34	-3.58	< 0.01

*Abbreviations: AITCS-II* Assessment of Interprofessional Team Collaboration Scale-version 2, *CCTDI* California Critical Thinking Disposition Inventory, *Pre* Pre-experiment, *Post* Post-experiment, *Z* Mann–Whitney U test values

### CCTDI Results post-intervention

Critical thinking subscale scores measured after the experiment are shown in Table 2. In general, critical thinking in the experimental group was higher than that of the control group, with statistically significant differences (*p* < 0.05). However, there was no statistically significant difference in the systematic ability subscore of the critical thinking scale between groups (*p* > 0.05).

**Table 2:** Post-Intervention CCTDI Scores

	Control group	Experimental group	Z	*P*
	Mean ± SD	Mean ± SD		
CCTDI (Total)	245.11 ± 30.56	284.08 ± 15.55	-5.81	< 0.01
Truth-seeking	35.53 ± 7.18	39.83 ± 3.67	-2.50	0.012
Open-mindedness	37.92 ± 6.02	42.47 ± 2.72	-3.86	< 0.01
Systematicity	39.50 ± 7.13	41.86 ± 5.19	-1.41	0.158
Self-confidence	30.03 ± 4.61	38.14 ± 4.26	-6.12	< 0.01
Inquisitiveness	32.06 ± 5.34	38.03 ± 4.23	-4.70	< 0.01
Maturity	39.33 ± 7.75	44.31 ± 3.58	-2.77	0.006
Analyticity	31.75 ± 5.40	38.44 ± 3.99	-4.96	< 0.01

*Abbreviations: CCTDI* California Critical Thinking Disposition Inventory, *Z* Mann–Whitney U test values

### Semi-structured interviews

The interviewer agreed that saturation had been reached after 13 interviews, thereby ending data collection. Interviews lasted between 37 and 45 min (mean duration of 41 min). Thirteen of the interviewees who were selected were nursing majors, including 7 in the experimental group and 6 in the control group. Interview results were summarized into three themes and two recommendations.

### Three themes

(1) Interprofessional combination can clarify role positioning: the roles of the members in the interprofessional portfolio team were clear, and they could perform their roles and responsibilities better by adhering to professional practice standards. Students from interprofessional teams said: “*I feel that the professional competencies required for each role have been met.*”*(E1) “I can find my position and make a difference.”(E2)* However, students in the same professional group said: *“Although I am playing Doctor, sometimes I still work as a nurse.” (C1)“I can't give professional advice.” (C2).*

(2) Interprofessional combination can improve team effectiveness. During interviews, students reported that interprofessional teams were more effective. Students from the interprofessional combination said: *“We work well together.”(E1) “We can deal with it quickly.” (E3) “Everyone is very professional, and the advice given is convincing.” (E5)* However, students from the same professional group said: *“Our cooperation was not smooth, and the simulation case was not running smoothly.” (C3) “Sometimes we can't persuade each other, so I choose to compromise.”(C6).*

(3) Interprofessional combination can enhance the learning experience. When talking about their learning experiences and feelings, the students in the interprofessional group gave more positive feedback. They believed that *“It is more interesting to cooperate with students from other majors in the classroom.”(E7) “I feel that this form is very novel, and I will try my best to make myself do better.”(E5) “I hope that other courses can also apply inter-professional teaching methods in the future.” (E4)* However, students in the same professional group said, *“I feel powerless when I play the role of a doctor.”(C4)* and *“I hope to adopt an inter-professional education method.” (C5).*

### Two suggestions

(1) Extend the time of the laboratory class. When asked about their ideas for improving the curriculum, the students wanted to extend the class hours and increase the number of simulated practice sessions. They said: *“I feel that five experimental classes are a little short.”(E1) “If time permits, I hope that each scene can be simulated twice.” (E3)“Because the first experimental class is the introduction to the course, the actual experimental training is only four times, which feels a bit less, so I hope that the number of times of experimental training can be increased.”(C3).*

(2) Simulated case scenarios should increase emergency department and community involvement. During the simulation scenarios, the students expressed the desire to add emergency departments and community hospitals to further improve their clinical critical thinking and teamwork ability to meet the practical needs of different scenarios. Their answers included: *“I think the emergency department has an important position in the hospital department and should be included.” (C2) “The emergency department has higher requirements for the independent judgment and problem-solving ability of medical staff, and I would hope to add this department.” (E4) “The country is currently vigorously developing community medical services, and community medical talents are also crucial, so I think this scene should be added.” (E7)“I plan to work in a community hospital in the future and hope to have an exercise in this context.” (E5).*

## Discussion

The results of the qualitative and quantitative analysis combined in this discussion showed that the use of SIPE as part of the a "Clinical Critical Thinking" curriculum improved students' clinical critical thinking and interprofessional cooperation abilities and cultivated their interprofessional core competencies. This may impact the teaching design of other courses in the future.

Critical thinking ability should be used as a mandatory indicator for evaluating the quality of nursing education at the undergraduate and higher levels [31]. While 67% of students in Smith et al. [32] study self-reported that interprofessional education improved their clinical thinking skills, critical thinking skills were not investigated. Our quantitative results showed that, with the exception of systematic ability, the total score and all subscores of critical thinking ability in the experimental group were significantly higher than those in the control group (*p* < 0.01). This result is consistent with the findings of Xie et al. [33]. Simulated interprofessional education has been shown to improve the clinical critical thinking of medical students. Aein et al. [34] showed that SIPE intervention can significantly improve the critical thinking ability of medical students, in particular their analytical reasoning and deductive reasoning. Simulation-based interprofessional medical education provides medical students with a safe and controllable clinical environment that allows for trial and error, the testing if judgmental hypotheses, and repeated practice exercises to expand clinical critical thinking [4, 8]. Three representative departments were examined in this study that could effectively improve the analytical ability of nursing students in different situations and enhance their self-confidence in the application critical thinking. The lack of statistical significance in the dimension of systematic ability between the two groups may be due to insufficient intervention time. Our qualitative results supported this, as the students expressed a desire for extended class hours. Teaching time will be increased in the future to study the effects of simulated interprofessional education on systematic ability.

Our quantitative results found that the interprofessional cooperative learning score of the experimental group was higher than that of the control group. This result indicates that the interprofessional cooperative team established an excellent cooperative partnership, demonstrated improved interprofessional cooperative learning ability, and actively coordinated and communicated to achieve teamwork goals, which are consistent with prior reports [35, 36]. Our qualitative results confirm this and explain the quantitative results to some extent. The working model for medical and nursing integration is being widely evaluated and applied in clinical practice. The integration of medical and nursing staff [37] occurs through joint planning, joint decision-making, joint goal-setting and problem-solving, and joint responsibility for patients through the cooperation of medicine and nursing. It requires mutual trust and respect on behalf of both parties.

Poor communication between physicians and nurses and poor teamwork are factors that hinder the integration of medical care, can negatively affect the quality and safety of nursing care, and may predispose adverse events [38]. This study established interprofessional learning teams to address low self-efficacy and low confidence in simulation practice with role-playing by a single professional. This team structure increased interest in learning, mobilized students’ subjective initiative, and jointly promoted the realization of team goals. This is conducive to establishing a relationship of mutual trust and respect between physicians and nurses in actual clinical practice in the future and promotes the development of an integrated medical and nursing work model.

Role ambiguity puts team members at risk of conflict, team dysfunction, and burnout [28]. The low team effectiveness of the control group in this study may have occurred because some team members played interprofessional roles. They may have lacked the professional ability and quality that matched their role and may also have been limited by their actual professional thinking, making their role orientation ambiguous. When team members encountered differences of opinion, they failed to give constructive opinions that fit their roles, causing other members to burn out. Finally, control group members chose a compromise method to help the team to reach a consensus. The interprofessional team structure of the experimental group made up for this deficiency and improved team effectiveness.

Communication is a central concept in interprofessional education and teaching. Effective communication promotes teamwork. Poor information transfer during medical practice is closely related to potential patient harm to patients and ultimately impacts treatment outcomes [39, 40]. The role experience of the experimental group may have been better than that of the control group because of their higher team effectiveness. The operating mode of the experimental group was closer to the integration of medical and nursing care. This group was responsible for overall patient care, could timely and accurately perceive patient needs, and apply appropriate treatments to make the teamwork goals faster and better. Communication plays a vital role in this. In contrast, the control group communicated poorly and took about twice as long to achieve the same effect. High-performing teams can increase team member self-confidence and enthusiasm for their work, making them more confident with solving problems.

In response to the suggestions from students to add emergency department scenarios to the cases, this study considers the emergency department’s high requirements for clinical critical thinking and teamwork ability when formulating the simulation’s teaching design. As class hours are limited, it may be difficult for students to achieve the ability requirements and thus no separate emergency department scenario was created. During the fifth week of the course, the instructor did not announce the case in advance and conducted a simulation demonstration by drawing lots on the spot to simulate an emergency scene as much as possible. The instructor did not consider that students wanted to add a community hospital scene to the case when designing the simulation case. Over the course of curricular reform the number of experimental courses will be increased, and more cases and scenarios will be available for simulation and demonstration.

This study represents several contributions to nursing and simulation study. First, in terms of research design, prior works derived their control and experimental groups from different grades [41]. The validity of the experimental results may have been influenced by the different educational backgrounds of students in different grades. This study recruited all participants from the same grade and the same point in medical education, thus reducing the influence of different educational backgrounds on experimental results. Second, prior works recruited the teaching team entirely from the same major [42]. The present work used a cross-major teaching team that made case setting more reasonable and closer to clinical practice. Third, in terms of research methods, most prior studies only employed modes of quantitative evaluation [43]. This study adopted a convergent mixed method that included both quantitative and qualitative components to explore research issues from different perspectives and to have a more comprehensive understanding of the study.

There are some limitations to this study. First, the sample size was small due to the limited enrollment of the schools included in this study. This may affect the reliability of the experimental results. Future works will include a larger sample size and will be conducted in schools with a larger number of students, thereby improving the credibility and promotion of the research results. Second, the implementation of interprofessional cooperation was limited. Due to insufficient publicity during the early stages of the study, we only recruited students majoring in clinical medicine and pharmacy to participate. Future works will increase publicity to attract students from more majors, such as psychology, rehabilitation, and nutrition to promote more in-depth interdisciplinary cooperation.

## Conclusion

This study used Biggs' 3P model to design a simulated interprofessional education course "Clinical Critical Thinking" and explore the effects of its application in China. We found that the SIPE model improved medical student critical thinking, interprofessional cooperation ability, and interprofessional core competence, and may serve as the basis for the design of other courses in the future.

## Data Availability

The datasets generated and analysed during the current study are not publicly available due we may have further research related to the data but are available from the corresponding author on reasonable request.
